# Endothelial-Specific EphA4 Negatively Regulates Native Pial Collateral Formation and Re-Perfusion following Hindlimb Ischemia

**DOI:** 10.1371/journal.pone.0159930

**Published:** 2016-07-28

**Authors:** Benjamin Okyere, Kaavya Giridhar, Amanda Hazy, Miao Chen, David Keimig, Robert C. Bielitz, Hehuang Xie, Jia-Qiang He, William R. Huckle, Michelle H. Theus

**Affiliations:** 1 The Department of Biomedical Sciences and Pathobiology, Virginia-Maryland College of Veterinary Medicine, Virginia Polytechnic Insititue and State University, 215 Duck Pond Drive, Blacksburg, Virginia, 24061, United States of America; 2 Virginia BioComplexity Institute, Virginia Polytechnic Insititue and State University, 1015 Life Science Circle, Blacksburg, Virginia, 24061, United States of America; University of Edinburgh, UNITED KINGDOM

## Abstract

Leptomeningeal anastomoses play a critical role in regulating vascular re-perfusion following obstruction, however, the mechanisms regulating their development remains under investingation. Our current findings indicate that EphA4 receptor is a novel negative regulator of collaterogenesis. We demonstrate that EphA4 is highly expressed on pial arteriole collaterals at post-natal day (P) 1 and 7, then significantly reduced by P21. Endothelial cell (EC)-specific loss of EphA4, EphA4^f/f^/Tie2::Cre (KO), resulted in an increase in the density but not diameter of pial collaterals compared to WT mice. ECs isolated from KO mice displayed a 3-fold increase in proliferation, enhanced migration, tube formation and elevated levels of phospho(p)-Akt compared to WT ECs. Attenuating p-Akt, using LY294002, reduced the proliferative and migration effects in the KO ECs. RNAseq analysis also revealed altered expression patterns for genes that regulate cell proliferation, vascular development, extracellular matrix and immune-mediate responses, namely MCP-1, MMP2 and angiopoietin-1. Lastly, we show that induction of hindlimb ischemia resulted in accelerated re-perfusion, collateral remodeling and reduced tissue necrosis in the absence of EC-specific EphA4 compared to WT mice. These findings demonstrate a novel role for EphA4 in the early development of the pial collateral network and suggests a role in regulating vascular remodeling after obstruction.

## Introduction

Leptomeningeal anastomoses, first described in 1874 by Heubner [1], are arteriole-to-arteriole anastomoses in the pia mater that connect the anterior (ACA), middle (MCA) or posterior (PCA) cerebral artery branches on both dorsal hemispheres. This naturally occurring adaptation can help restore blood flow to vascular territories downstream of an occluded artery by providing retrograde perfusion. Leptomeningeal anastomoses constitute an important ‘by-pass’ system that provides an alternate route for oxygen, nutrients and potential therapeutic agents. Clinically, patients having greater numbers of pre-existing collaterals in skeletal muscle, heart and brain recover better following vascular occlusion [2–8]. Similarly, collateral density varies widely amongst different strains of mice and dictates their outcome from vascular occlusion [9–12]. While an extensive, functional collateral network is inextricably linked to neuroprotection and is an undisputed target for therapeutic intervention, the mechanism(s) underlying native collateral formation remains poorly understood.

Collateral vessel formation arises through a series of orchestrated events. Recently it has been described that pial collaterals begin to form at embryonic day 15.5 (E15.5) in mice, after the MCA has extended across the cerebral cortex [13]. This occurs after the formation of the primary vascular plexus where endothelial cells are mainly dividing, branching and migrating along astrocytes. Interestingly, astrocytes provide the main cues for endothelial cell guidance [14] during this time, similar to that in retinal vascular development [15–17]. The establishment of the pial collateral network peaks at E18.5 to post-natal day 1, followed by significant post-natal pruning which is complete by P21 [13]. The pial collateral density, which is set during embryonic development, is strain specific and these strain differences are maintained into adulthood [13]. Furthermore, embryonic perturbation of collateral formation results in life-long changes in collateral density [18]. These findings indicate that genetic factors largely dictate collateral establishment and that pre-natal exposures may influence their development. Although the genetic polymorphisms which create strain-specific variations in collateral numbers are unknown, vascular endothelial growth factor (VEGF), A disintegrin and metalloprotease Family Members (ADAM) 10 and 17 as well as chloride intracellular channel (Clic4) have been shown to regulate collateral density and diameter during development as well as remodeling after ischemia [9, 18–20].

Our current findings indicate that EphA4 receptor is a novel negative regulator of pial arteriole collateral formation in the brain. Eph receptors are widely known to control cell migration, proliferation, boundary formation and repulsive/attractive cues [21–25]. Within the CNS, they regulate axonal guidance and fasciculation, neural crest migration, midline development, and synaptic plasticity [26–32]. Eph receptors are the largest known family of transmembrane receptor tyrosine kinases which contain 14 members [26, 33] and are divided into two distinct subfamilies, EphA and EphB, both of which bind to *membrane-anchored ephrin ligands* and require cell-cell interaction. The EphA receptors bind to ephrin-A ligands that are anchored to the membrane by a glycosylphosphatidylinositol linkage, while the EphB receptors interact with three related transmembrane-spanning ephrin-B ligands [24]. There is some promiscuity between subclasses, such as EphA4, which has been shown to bind with high affinity to both the A-class and B-class ephrins. This broad overlap of binding specificities within these subclasses suggests the possibility of compensatory activity and redundancy among family members. The involvement of Eph signaling in tumor and vascular development is well established for several family members [34]. For example, ephrinB2 and EphB4 knockouts show defects in arterio-venous patterning [35] and the use of soluble recombinant ephrinB2 or EphB4 proteins can affect migration, adhesion and proliferation of cultured ECs and tumor angiogenesis [21, 36–38]. In addition, both ephrinB1 and ephrinA1 ligands have been shown to positively regulate angiogenesis [39, 40]. Morphological differences in the vasculature of the spinal cord and hippocampus has also been recently described in EphA4- and ephrinA5-defecient mice, respectively [25, 41]. Although ephrin/Eph interactions have been deomonstrated during neural, cardiac, retinal and cancer development, few studies have addressed their role in *cerebral* arteriole development and whether this may influence tissue outcome after vascular obstruction.

The current study addresses the role of EphA4 in pial collateral formation using a cell-specific gene targeted approach. We find that endothelial cell (EC)-specific ablation of EphA4 significantly enhanced the presence of leptomeningeal anastomoses which reflects an increase in the proliferation and migration of EphA4-null cultured ECs. We also show that p-AKT mediates these effects and is negatively regulated by EphA4 resulting in suppression of proliferation, migration and expression of key vascular proteins. Assessment of blood flow, collateral growth/remodeling and tissue necrosis following hindlimb ischemia also reveals substantial improvements in perfusion, collateral enlargement and appearance scoring in EC-specific EphA4 knockout mice. These findings indicate that EphA4 negatively regulates pial collateral formation in a cell-specific fashion and plays a key role in the peripheral vascular response to obstruction.

## Results

### Post-natal expression of EphA4 on pial arteriole collateral vessels

**Fig 1:**
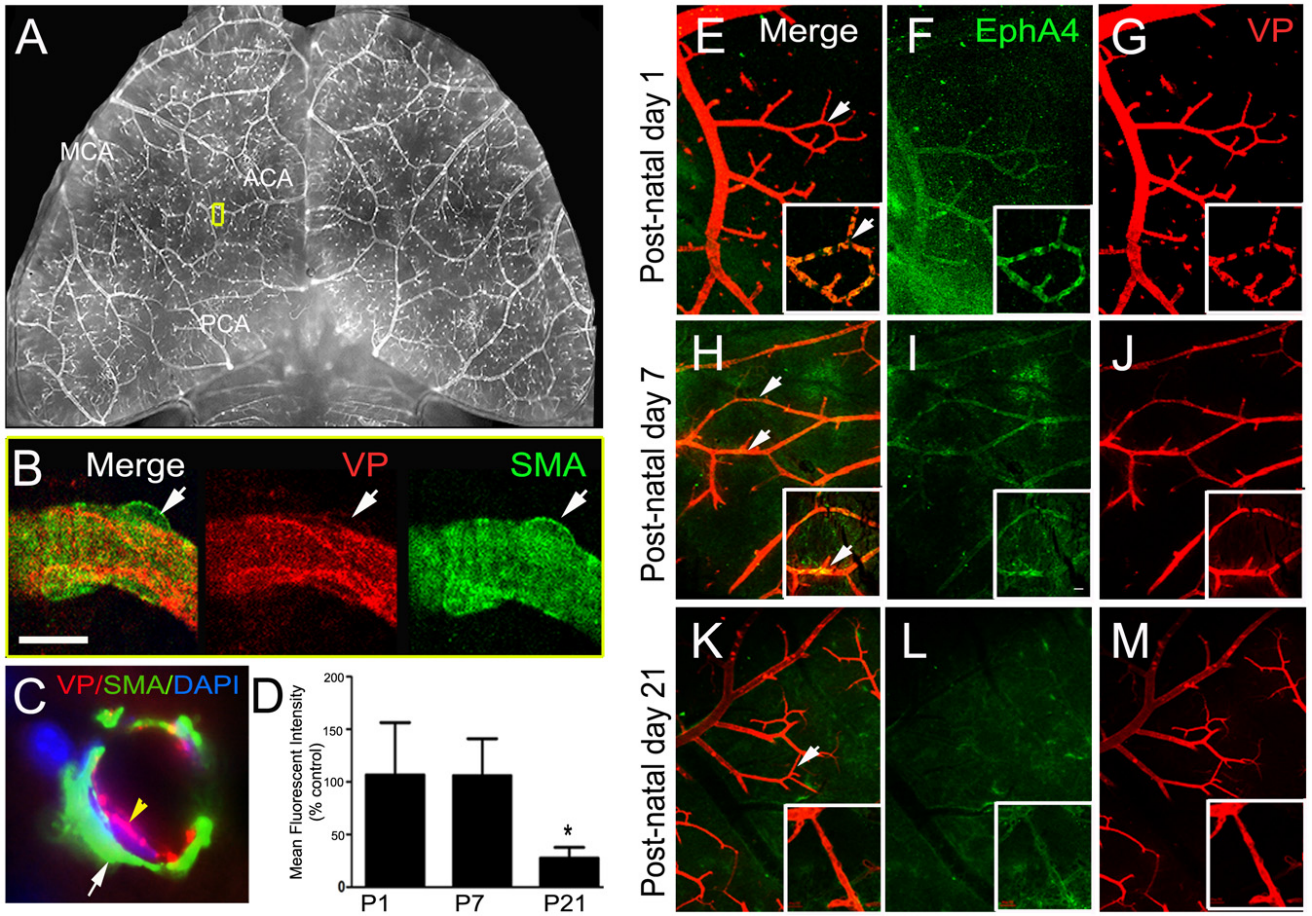
Vessel Painting and co-labeling of post-natal murine pial vessels. (A) Inverted VP image of a mouse brain showing selective labeling of the cerebral arteriole network using vessel painting. (B) Identification of pial collaterals using vessel painting (VP, red) and double labeling of with anti-SMA shows, VP does not label SMCs. (C) Confocal image showing cross section a vessel painted pial arteriole, immunotained for SMA, confirming that VP does not label SMCs. (D) Quantification of EphA4 on pial collaterals, represented as mean fluorescence intensity. (E—M) Double labeling of psot-natal day (P)1, P7 and P21 brains using VP (red) and anti-EphA4 (green). EphA4 is expressed on VP-labeled collaterals at P1, P7 and minimally at P21. *P<0.05; n = 5 per group; represented as mean ± SEM.

Previous studies have shown EphA4 to be expressed on platelet endothelial cell adhesion molecule-1 (PECAM/CD31)-positive cells in the cortex at E16, down regulated in adulthood and upregulated on astrocytes and ECs after CNS injury [25]. To investigate the role of EphA4 in arteriole collateral formation we first assessed its expression on pial arterioles using vessel painting (VP), *which selectively labels the adult arteriole vasculature*, following transcardial Dioctadecyl-Tetramethylindocarbocyan (Dil) infusion [42]. We modified this technique in order to visualize and assess post-natal (P1-P21) pial arteriole development. Post-natal pial collateral vessels are strongly labeled following VP (Fig 1A and 1B) whereas the associated smooth muscle cells, identified by smooth muscle actin (SMA), do not co-label with the Dil stain (Fig 1A, 1C and 1D; high mag confocal image). Using immunofluorescence labeling and confocal image analysis on whole mount cortical tissue from post-natal day 1, 7 and 21, we found EphA4 to be highly expressed on pial arteriole collaterals at P1 (Fig 1E–1G) and P7 (Fig 1H–1J) then reduced by P21 (Fig 1K–1M). Quantified expression analysis was performed at P1-P21 (n = 3/group) for EphA4 using immunohistochemistry and densitometry which demonstrated a significant reduction in EphA4 immunoreactivity on VP-labeled pial collaterals (Fig 1N). This is the first demonstration of Eph/ephrin expression on the pial cerebral arteriole network.

### Endothelial-specific ablation of EphA4 increases the presence of pial arteriole collateral vessels

**Fig 2:**
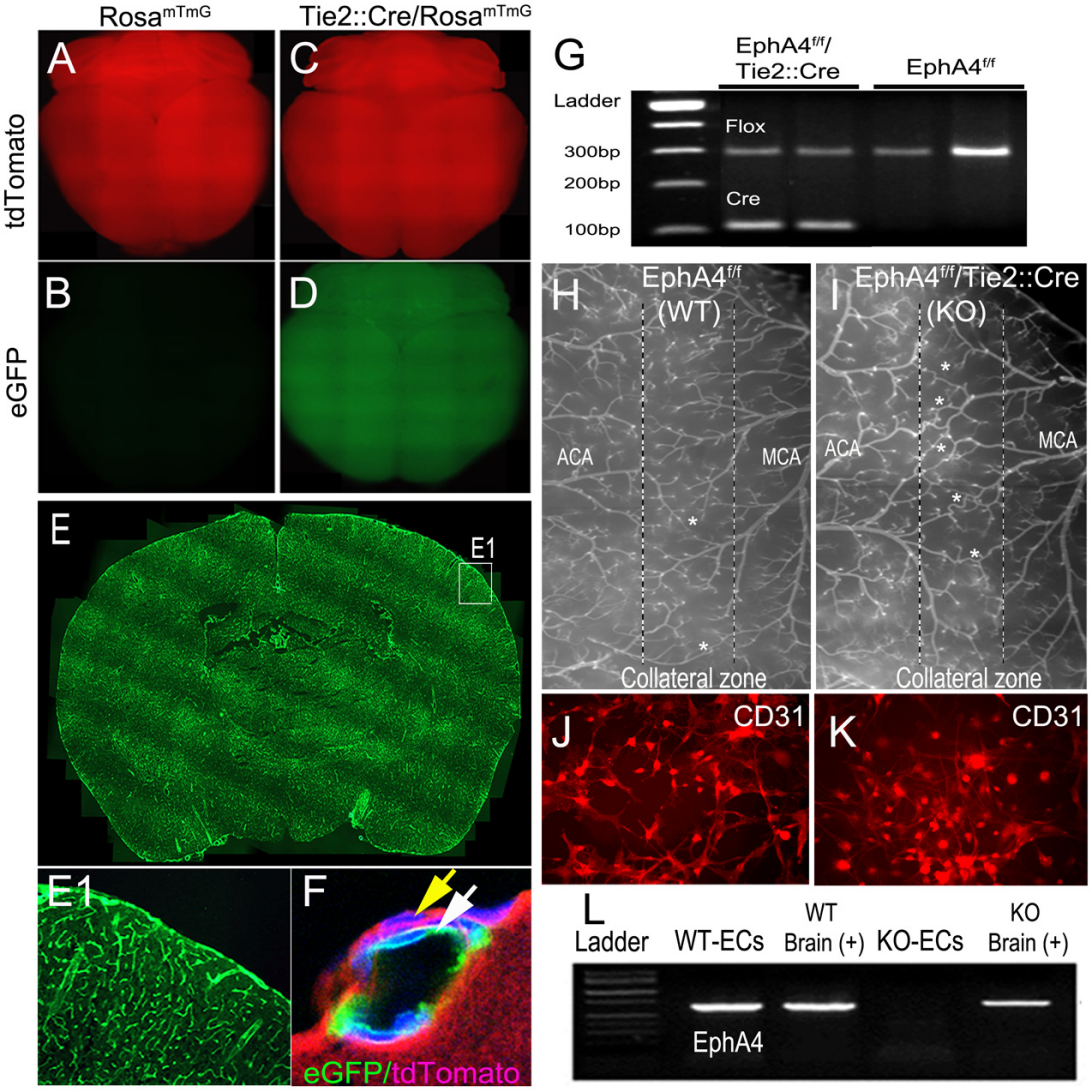
Phenotype and genotype of littermates from EphA4f/f and Tie2::Cre breeding paradigm. (A—F) Reporter labeling of Tie2::Cre is specific to blood vessels. Panel F further confirms eGFP expression specifically to blood vessels. (G) Genotyping using PCR shows a flox primers of about 290bp and a bottom Cre band. Lane 1 and 2: EphA4^f/f^/Tie2::Cre (KO); Lane 3 and 4: EphA4^f/f^ (WT). Vessel painting of surface pial arterioles of WT (H) show increased numbers in KO (I) mice. Panel J and K are WT and KO endothelial cells cultured from brain endothelial cell progenitor cells. ECs are labeled with CD31 (green) and DAPI (blue). (L) ECs from KO mice do not express EphA4 RNA transcripts.

Due to the widely expressed and complex nature of EphA4 in CNS development and injury-induced responses [25, 43, 44], we utilized a cell-specific approach to investigate its role in pial collateral development. We generated conditional knockout mice using the loxP/Cre system driven by the Tie2 promotor, which has been shown to be selectively expressed in endothelial cells (EC) [45]. To demonstrate EC specificity, we bred Tie2::Cre mice with Rosa^mTmG^ double-reporter mice which expresses tdTomato (mT; Rosa^mTmG^) prior to Cre-mediated excision (Fig 2A and 2B) and GFP (mG; Tie2::Cre/Rosa^mTmG^) after excision (Fig 2C and 2D) [46]. Coronal sections from Tie2::Cre/Rosa^mTmG^ mice were subjected to confocal microscopy in order to visualize exclusive GFP expression in Tie2::Cre-positive vessels in the pial surface (Fig 2F; white arrow), surrounded by tdTomato-positive support cells (Fig 2F; yellow arrow). Next, we generated EphA4 EC-specific, Tie2::Cre/EphA4^f/f^ knockout mice (KO) and EphA4^f/f^ wild type mice (WT) [47], which carry the homozygous floxed alleles and Cre recombinase or floxed allele only, respectively (Fig 2G). Adult WT and KO mice were then analyzed for the presence of pial collaterals using vessel painting. In the absence of EC-specific EphA4, we find greater numbers of pial collaterals between the middle cerebral artery (MCA) and anterior cerebral artery (ACA) branches (Fig 2I compared to 2H). WT and KO ECs were then isolated and cultured from whole pup brain tissue of EphA4^f/f^ and Tie2::Cre/Rosa^mTmG^ mice using CD31 microbeads. Both WT and KO brain-derived ECs express CD31 [48] (Fig 2J and 2K, respectively) while mRNA transcripts for EphA4 are present in WT and absent in KO ECs (Fig 2L). These findings demonstrate that EC-specific ablation of EphA4 enhances the density of pial arterioles in the adult murine brain.

### Post-natal time course of pial arteriole collateral formation following EC-specific EphA4 ablation

**Fig 3:**
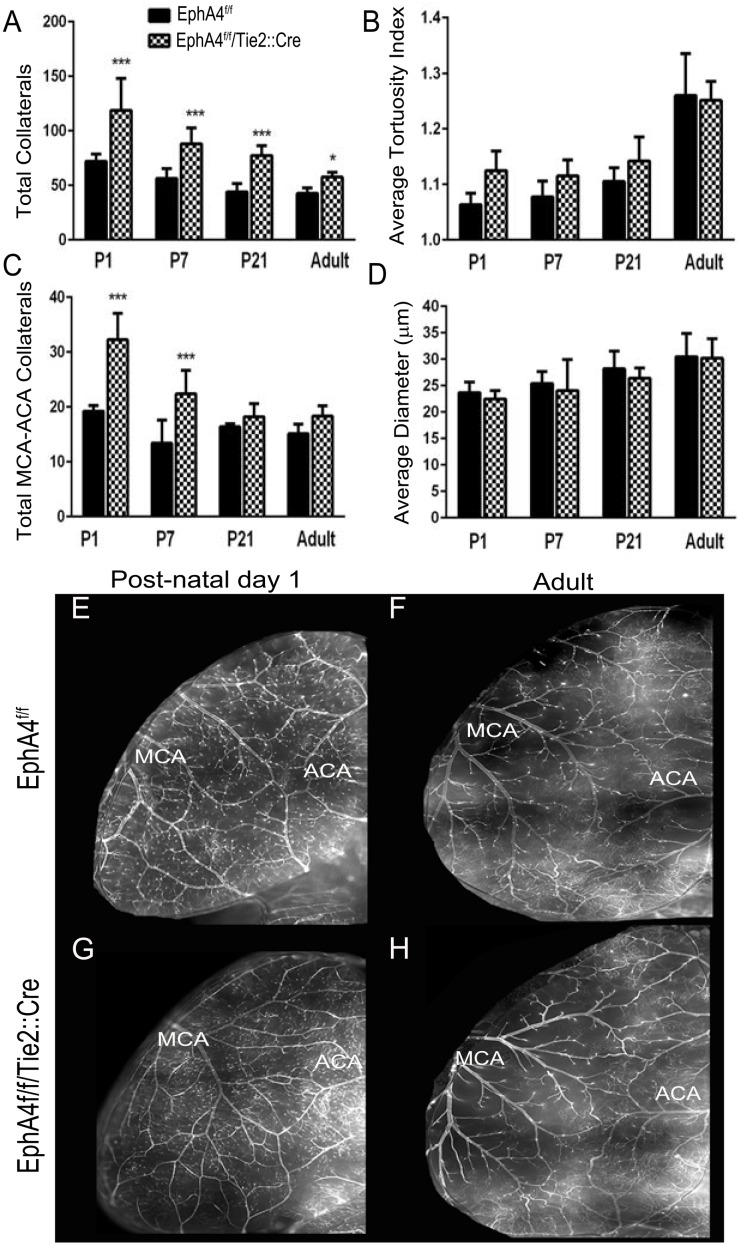
EphA4 EC-specific ablation increases collateral formation in the post-natal murine brain. (A) KO mice have increased total collaterals in P1, P7, P21 and adult mice. (B) average tortuosity is increased in all KO groups except adult. (C) KO mice have more MCA—ACA collaterals but there is no significant difference in average diameter of collaterals in the mice. (E—H) VP of P1 and adult WT and KO brains. *P<0.05; ***P<0.001; n = 5–9 per group; represented as mean ± SEM.

Recent studies have shown that pial collaterals are initially formed and peak during pre-natal development (E14-birth,) after which time there is a significant post-natal pruning process (P1-P21) followed by maturation of the collaterals (P21-adult) [13]. To determine whether EphA4 limits collateral development by regulating these discrete phases, we modified the vessel painting technique to stain for post-natal pial collaterals at P1, P7 and P21. Analysis of the total number of collaterals show that KO mice (Fig 3A, 3G and 3H) display a significantly higher number of vessels at P1 (118.8 ± 14.6 vs. 72 ± 2.9), P7 (88 ± 5.1 vs. 56.4 ± 4.0), P21 (77.4 ± 3.9 vs. 44.0 ± 3.4) and adult (57.6 ± 1.4 vs. 42.6 ± 1.5) stages compared to WT mice (Fig 3A, 3E and 3F). No differences were seen in the tortuosity index (Fig 3B) or diameter (Fig 3C) of the collaterals during the post-natal and maturation stages. Smooth muscle cell coverage was also not affected on pial collateral vessels following EphA4 ablation at P1-P21 as observed using whole mount anti-SMA staining (data not shown). Interestingly, the extent of post-natal pruning is similar between WT and KO suggesting that pre-natal expression of endothelial cell (EC)-specific EphA4 plays a critical role in suppressing initial pial collateral formation.

### EphA4 limits EC proliferation, migration and tube formation via PI3K/Akt suppression

**Fig 4:**
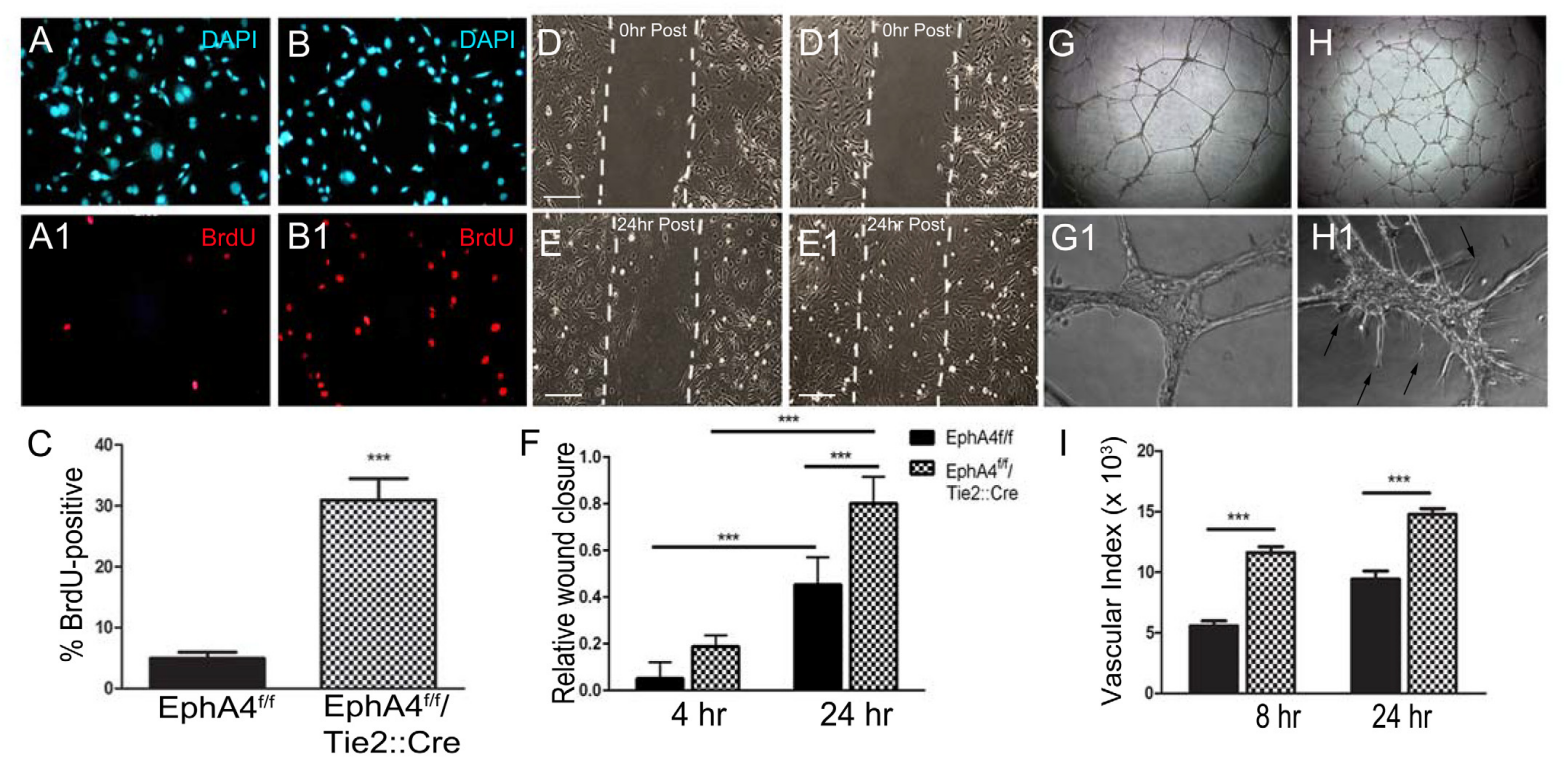
EphA4 EC-specific ablation increases EC proliferation, migration and tube formation. Panel shows WT (A, A1) and KO (B, B1) BrdU analysis of ECs stained with DAPI (blue) and BrdU (red). (C) KO ECs have significantly increased BrdU incorporation compared to WT ECs. WT (D, D1) and KO (E, E1) EC scratch. (F) KO have greater migration rate 24 hours post scratch compared to WT EC. (G, G1) WT and (H, H1) KO ECs were seeded on growth factor-reduced matrigel to form tubes. (I) Vascular index (number of tubes x tube length) is enhanced in KO ECs compared to WT. ***P<0.001; n = 16–20 wells/group; represented as mean ± SEM.

Endothelial cell proliferation during this time has been shown to be critical in establishing the pial collateral network [13, 19]. EphA4 may therefore suppress EC expansion to regulate pre-natal collateral formation. To examine this possibility, we analyzed proliferation, migration and tube formation of cultured ECs derived from the post-natal day 1 brains of EphA4^flox/flox^/Tie2::Cre (KO) and EphA4^flox/flox^ (WT) mice. Using bromodeoxyuridine (BrdU)-labeling, we found a significant increase in the percentage of BrdU-positive KO-ECs (30.95 ± 3.53; Fig 4B, 4B1 and 4C) after 24 hrs of culture, compare to WT-ECs (4.9 ± 1.02%; Fig 4A, 4A1 and 4C). Next, we assessed migration using an *in vitro* wound closure or scratch assay [49]. Under growth factor-free conditions, KO ECs display an increase rate of wound closure (0.8 ± 0.05 relative to post-scratch) (Fig 4D1 and 4F) compared to WT ECs (0.45 + 0.04 relative to post-scratch) (Fig 4E1 and 4F) at 24 hrs post-scratch. Upon plating onto matrigel substrate, ECs attach and degrade the surrounding extracellular matrix to create guidance pathways that facilitate migration and tube formation [50]. In the absence of EphA4, ECs display an increase in vascular index (number of tubes x tube length) during tube formation (Fig 4H and 4I) compared to WT ECs (Fig 4G and 4I) at 8 and 24 hrs after plating. KO ECs also displayed greater numbers of cells that appeared to sprout from the original formed tubes, which usually became apparent at 24 hours after plating (Fig 4H1). These findings demonstrate that EphA4 plays a central role in suppressing the expansion and migration of brain-derived endothelial cells, which may influence the extent of collateral formation during critical periods of development.

**Fig 5:**
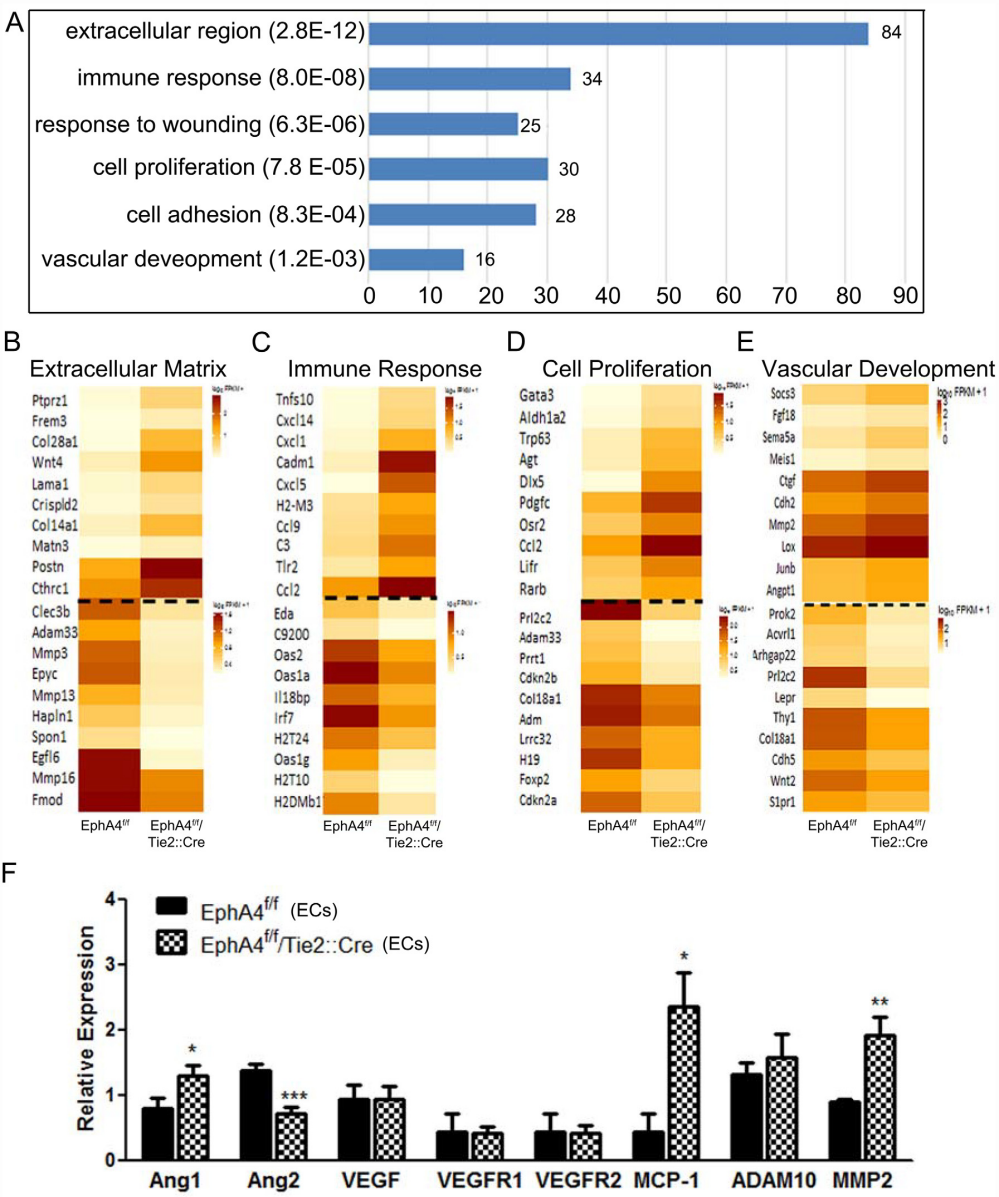
mRNA sequence profile of WT and KO ECs. (A) Genome-wide differential expression analysis via RNA-sequencing indicates KO ECs differening gene expressions that predominantly effect the extracellular matrix, immune response, wound healing, cell proliferation, cell adhesion and vascular development. (B—E) Overall ontological analysis of both upregulated and downregulated genes between WT and KO ECs. (F) Differential expression of seven genes significant in vascular development was further assessed using qRT-PCR which confirms an increase in Ang-1, MCP-1, and MMP2 in KO RNA transcripts using PCR. *P<0.05; **P<0.01; represented as mean ± SEM.

Next we sought to identify key downstream mediators of EphA4 signaling in ECs. We isolated RNA from WT and KO ECs then assessed differences in global transcript levels using RNAseq analysis. Genome-wide differential expression analysis via RNA-sequencing showed significant changes in gene expression for EphA4-null ECs. Gene ontology analysis of significantly upregulated genes (≥2-fold expression change, p value ≤0.01) using DAVID found that knockout cells show increased expression of genes with products residing in the extracellular region as well as upregulation of genes involved in the immune response, cell proliferation and adhesion, and vascular development (Fig 5A). Overall ontological analysis of both upregulated and downregulated genes also found that knockout cells display significantly altered expression patterns for genes relevant to the extracellular matrix, immune response, cell proliferation, and vascular development (Fig 5B–5E). Based on the results of gene ontology analysis, differential expression of seven genes significant in vascular development was further assessed using qRT-PCR (Fig 5F). This assessment confirmed significant upregulation of Ang1, MCP1 and MMP2 downregulation of Ang2, as previously indicated by sequencing data.

**Fig 6:**
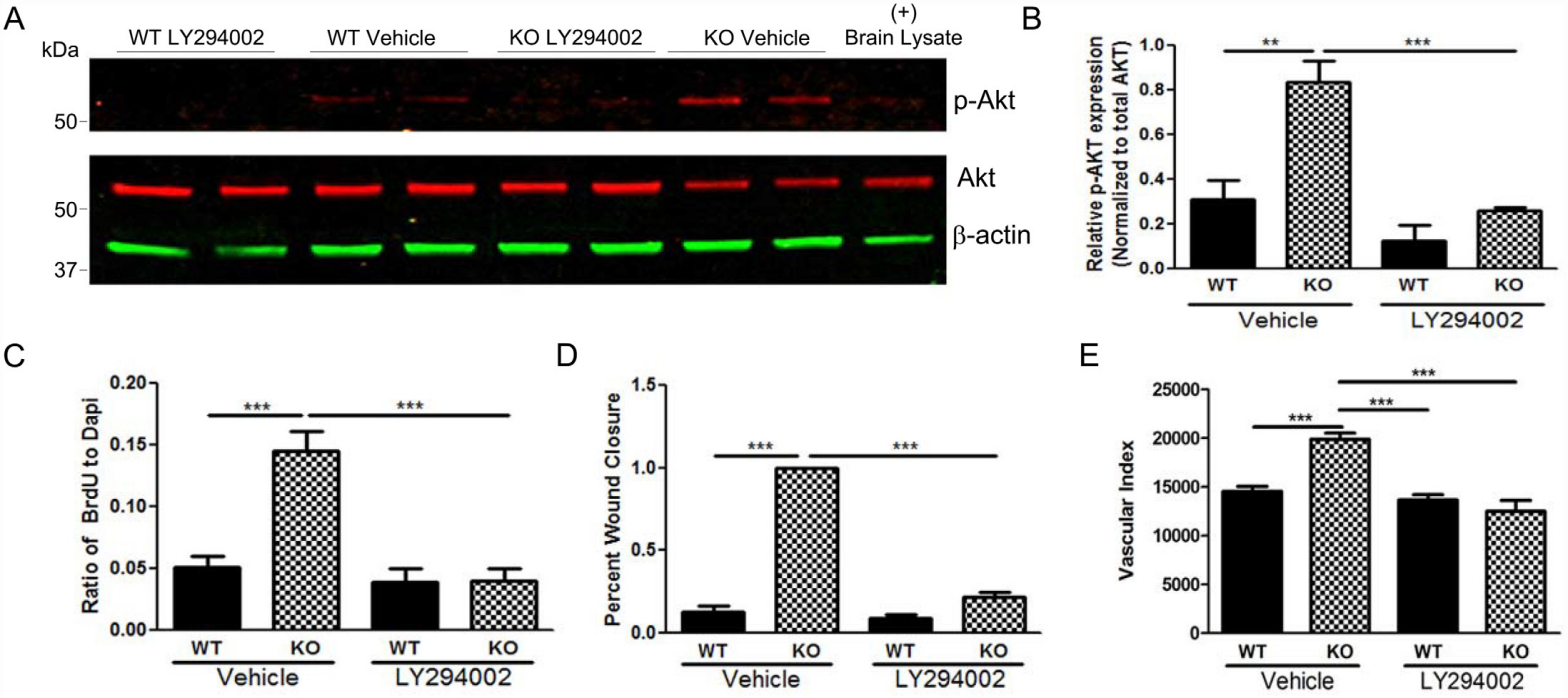
PI3K inhibition attenuates endothelial cell functions following EphA4 ablation. (A) Western blot analysis indicates increased p-Akt expression in KO ECs compared to WT ECs. Expression is reduced in KO ECs after LY294002 treatment. (B) Densitometric analysis shows a three-fold increase of p-Akt in KO ECs. KO treated with LY294002 have identical p-Akt expression as WT vehicle control. (C—E) In addition treating KO ECs with LY294002 significantly reduced BrdU incorporation, migration and vascular index, respectively. ***P<0.001; n = 16–20 wells/group; represented as mean ± SEM.

We further assessed the role of the PI3K/Akt pathway in mediating EphA4 effects in cultured ECs. Protein lysates were collected from WT and KO ECs following 24-hour vehicle or PI3K inhibitor LY294002 treatment, then analyzed for phospo-Akt (p-Akt) by WesternBlot. P-Akt was significantly up-regulated in vehicle treated KO ECs (Fig 6A and 6B) compared to WT, which correlated with increased proliferation and migration. LY294002 significantly reduced p-Akt expression levels in KO ECs. Inhibition of p-AKT resulted in attenuation of proliferation (Fig 6C) and migration during scratch wound closure and a trend towards reduced tube formation (Fig 6D and 6E, respectively) in KO compared to WT ECs. These data indicate that EphA4 supresses p-AKT which limits proliferation and migration of endothelial cells.

### EC ablation of EphA4 increases hindlimb collateral circulation and remodeling after femoral artery occlusion

**Fig 7:**
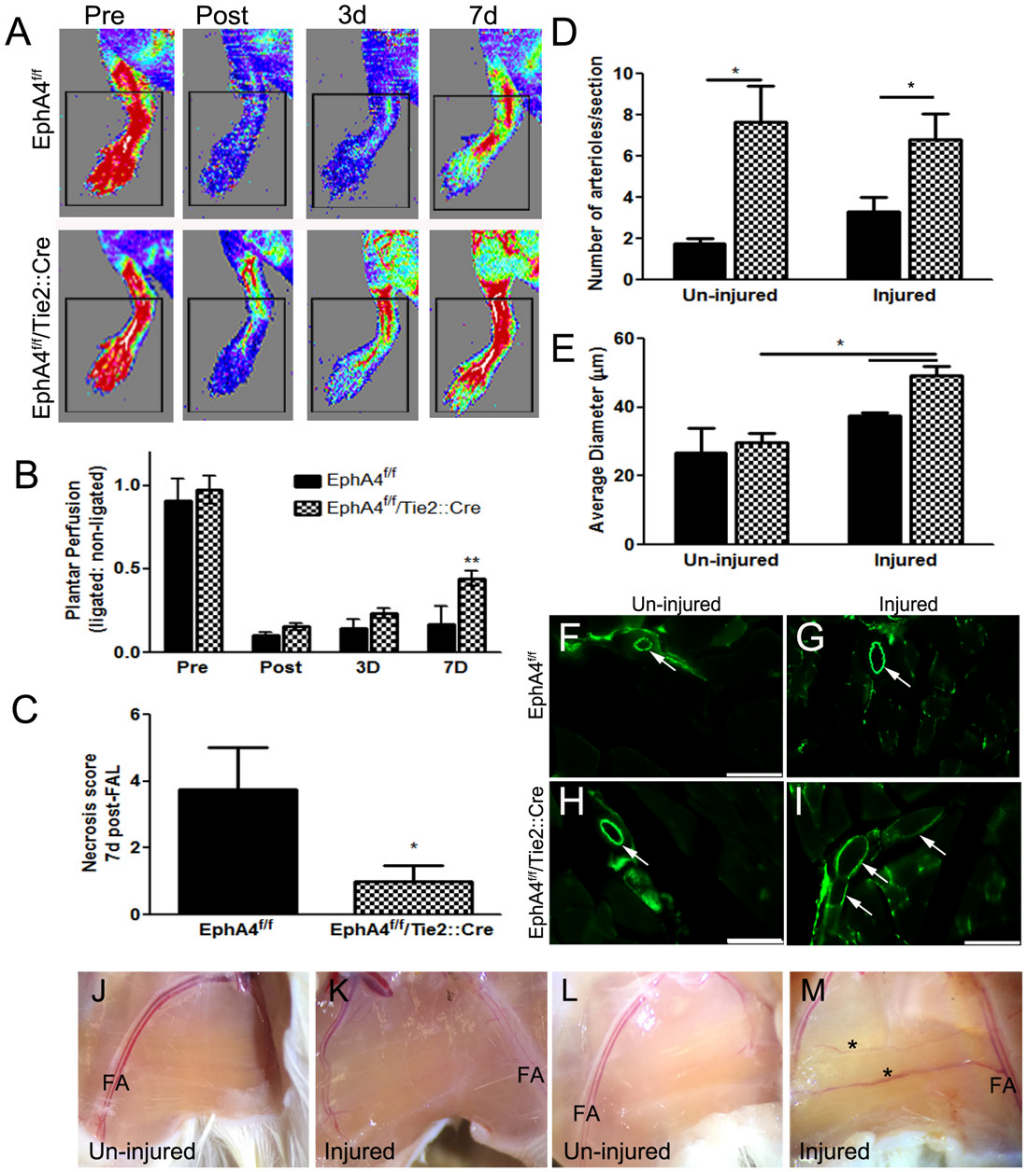
Plantar reperfusion and collateral remodeling post- hindlimb ischemia. (A) Laser Doppler images of blood flow pre and post ischemia in WT and KO mice. (B) Analysis shows significant increase in plantar blood flow perfusion at 7d post hindlimb ischemia or femoral artery ligation (FAL) in KO mice compared to WT. (C) KO mice also have a significant reduction in toe necrosis compared to WT mice at 7 days post-FAL. (D) The number of CD31-positive arterioles were are in increased in serial sections of both un-injuured and injured adductor muscles in the absence of EC-specific EphA4. (E) Average arteriole diameter in the injured aductor muscle is increased in KO mice compared to WT and KO un-injured tissue at 7d post-FAL. (F-I) Representative high magnification (x20) images from un-injured and injured adductor muscles at 7d post-FAL using CD31 immuno-staining and -fluorescence. Scale bar = 100μm. (J-M) Brightfield images of WT (J and K) and KO (L and M) adductor muscles at 7d post-FAL. *P<0.05; **P<0.01; n = 5-8/group; represented as mean ± SEM. FA = Femoral artery.

The extent of preexisting collaterals and the capacity of these vessels to remodel (enlarge) are major determinants of tissue injury following obstruction. The variation in collateral density within the hindlimb circulation as well as in vascular branching in the retina of different mouse strains has been shown to be consistent with that of the pial network [11, 12, 20, 51]. Therefore, to address whether the density and/or remodeling of the collateral circulation in EC-specific EphA4 knockout mice correlates with tissue perfusion and ischemic outcome, we utilized the hindlimb ischemia model by performing femoral artery ligation (FAL) in adult WT and KO mice. We measured recovery of hindlimb plantar perfusion up to 7 days post-FAL using high-resolution laser Doppler imaging and performed necrosis scoring (number of necrotic toes). Plantar perfusion (index of leg perfusion) was reduced more immediately after FAL in WT mice compared to KO mice and recovered faster in KO at 3 and 7 days, although statistical significance was not reached until day 7 (Fig 7A and 7B). Toe necrosis was also significantly reduced in KO compared to WT mice at 7 days post-FAL (Fig 7C) which correlates with greater re-perfusion. Serial sections of the injured and un-injured adductor muscles were stained for CD31 and the number and diameter analyzed. The adductor muscle from KO mice display greater numbers of arterioles both in the injured and un-injured tissue (Fig 7D) compared to WT mice. In addition, their size is significantly increased in the injured vs. un-injured limb (Fig 7I and 7M vs 7H and 7L, respectively) compared to WT (Fig 7G and 7K vs 7F and 7J, respectively) suggesting restoration of blood flow and reduced tissue necrosis may result from greater collateral density and diameter following FAL. The capillary density in the adductor muscle was not significantly different between the two groups (data not shown). The density and diameter of pre-existing collaterals in the adductor thigh dictates acute plantar perfusion immediately following FAL [9, 11, 12] whereas outward remodeling of their lumen diameter (i.e., arteriogenesis) governs recovery of perfusion days to weeks post-occlusion [12, 52]. Thus, our findings indicate that plantar perfusion and tissue recovery is negatively regulated by EC-specific EphA4.

## Discussion

Leptomeningeal anastomoses represent a prominent structural feature in the pial surface of the brain. Although inactive under normal physiological conditions, shear stress following vascular occlusion induces collateral outward growth and remodeling which provides an alternate route for blood flow and perserves tissue function. This important adaptive response varies amoung individuals and is density dependent. Differences in the abundance of native pial collaterals also exists between different strains of mice, which is attributed to natural polymorphisms and is age-dependent [9, 10, 12, 51, 53–55]. An expansive pre-existing collateral network is deemed favorable for patient outcome following vascular occlusion [56, 57], however, the key players involved in the development of this network remain unclear. Using a cell-specific transgenic approach, our current findings demonstrate that EphA4 ablation on endothelial cells increases the number of pial arteriole collaterals. This effect was prominent as early as post-natal day 1, indicating that EphA4 limits their pre-natal formation which peaks at embryonic day 18-P1[13]. Post-natal pruning, however, was unaltered in the EphA4^flox/flox^/Tie2::Cre (KO) compared to EphA4^flox/flox^ (WT) mice which further suggests that EphA4 negatively regulates an early embryonic event resulting in robust collateral formation upon EC-specific deletion. Smooth muscle cells (SMC) are also an integral part of collateral function [58–62]. Although we did not observe any difference in SMC coverage in the post-natal collateral, we cannot rule out any indirect effects of EC ablation of EphA4 on SMCs. Given that endothelial-to-smooth muscle cell junctions in collateral vessels have never been identified [63, 64], as they are separated by internal elastic lamina, we do not believe they play a major role during pre-natal collaterogenesis in the absence of EphA4

Increased EC proliferation during primary vascular plexus formation has been attributed to an increase in collateral number [18]. Our findings indicate that EphA4 significantly limits EC division by suppressing phosphorylation (p) of Akt. Attenuation of p-Akt using LY29004 in KO ECs reversed the proliferative effects. Likewise, EC migration was also restricted by EphA4 following scratch injury and during vascular tube induction. Preventing p-Akt attenuated the migration of KO ECs to WT levels during wound closure and tube formation. Upon plating onto matrigel substrate, ECs attach and degrade the surrounding extracellular matrix to create guidance pathways that facilitate migration and tube formation [50]. Our data suggests that EphA4 restricts key players in this process, namely, PI3K/Akt, angipoeitin-1, MCP-1 and MMP2 [65, 66]. Of note, the PI3K/Akt pathway has been shown to stimulate EC proliferation, migration and tube formation downstream of VEGFR [67–69] and angiopoeitin signaling [66, 70–73], known mediators of collateral formation and remodeling [74, 75], respectively. Previous findings have demonstrated that Eph receptor signaling directly suppresses p-Akt upon ligand stimulation [76]. EC-specific p-Akt may represent a major determinant in native collateral formation by balancing the signals between negative EphA4 and positive Flk-1 signaling [9, 19]. While we show EphA4 does not directly regulate expression of VEGF or VEGFR1/R2, EphA4 may limit the known functions of VEGFR signaling indirectly by limiting downstream Akt phosphorylation to suppress proliferation, migration and vascular morphogenesis. Indeed, paracrine VEGF-stimulation of Flk-1-notch signaling on ECs was shown to positively regulate embryonic collaterogenesis [18]. Adam 10 and Adam 17 were also implicated in this process. Our findings did not reveal a difference in expression of either Adam 10 or 17 suggesting EphA4 may regulate collateral formation though a novel pathway involving Akt. Interestingly, while angiopoietin-1 appears to play a critical role in regulating vessel branching during development [77, 78] as well as in stimulating remodeling [79–82] and cytokine and chemokine induction, including MMPs, MCP-1, CXCL-5, -10, -9 and MMP-2, -9 [83–86], its role in collaterogenesis has not been established. Recently, using the Cre-lox system, angiopoietin-1’s role in regulating the vascular response to injury was confirmed [77]. Given the embryonic lethality of germ-line deletion, floxed angiopoietin-1 mice may be useful for future studies investigating its potential cell-specific role in pial collateral formation.

The density and diameter of pre-existing collaterals in adult tissue, as well as their ability to enlarge (remodel) following arterial occlusion dictates the severity of ischemic injury. While we have elucidated a novel mechanism regulating collateral formation, it is unclear whether EphA4 is a key player in adult collateral remodeling post-obstruction (arteriogenesis). Using a well-established model of hindlimb ischemia, we find significant recovery of hindlimb blood flow within a week following artery ligation which correlated with reduced toe necrosis in EC-specific EphA4 knockout mice. Unlike sprouting angiogenesis, which is incapable of fully restoring the function of larger damaged vessels [87], arteriogenesis or outward growth of pre-existing collaterals [88–90] can result in an expeditious return of blood flow. They also carry ten times more blood volume compared to capillaries and therefore have a much greater capacity to prevent tissue loss. The quick return of plantar perfusion observed in the absence of EC-specific EphA4 after hindlimb ischemia suggests that arteriole remodeling may also be negatively regulated by EphA4. Currently it is unclear what major role EphA4 may regualate in this process, however, on-going studies are elucidating its role in several aspects of the remodeling process. Initiation of arteriogenesis occurs due to changes in fluid shear stress which activates the endothelium leading to recruitment and proliferation of vascular and peri-vascular cell types as well as reorganization of the extracellular matrix, all of which are coordinated through a temporal pattern of cytokine, chemokine, growth factor, and protease expression. The EC is the first to respond by sensing this change through mechano-transduction leading to transcription of downstream gene targets necessary for growth and remodeling [91–95]. It is possible that EC-specific EphA4 could regulate some aspects of the mechano-transduction pathway leading to modulation of EC proliferation and immune cell recruitment. Future studies will address this important process.

The current investigation highlights a novel suppressive pathway involved in collateral formation during development, which focuses on the cell autonomous role of EphA4 using EC-specific knockout mice. EphA4 limits the proliferation and migration of ECs via p-Akt regulation, which may play a substantial role in pre-natal collateral formation. EphA4 also negatively regulates EC expression of angiopoietin-1, MCP-1 and MMP2 which may have a profound effect on collateral density during development and/or remodeling following ischemia. Future studies will evaluate th role of EphA4 on collateral remodeling in the adult brain following occlusion and the specific mechanism(s) driving the remodeling events using our EC-specific approach.

## Materials and Methods

### Animals

All mice were generated and housed in an AAALAC approved, virus/antigen-free facility with a 12 h light-dark cycle; food and water *ad libitum*. We employed a recently generated mouse strain carrying a LoxP-flanked (floxed) EphA4 gene (EphA4^flox/flox^) (Jackson Labs, Epha4^tm1.1Bzh^/J) [47]. EphA4^flox/flox^ mice were bred to transgenic mice expressing Cre recombinase under the direction of the tyrosine kinase Tek (Tie2) promoter/enhancer (Tie2::Cre^tg/+^) (Jackson Labs, B6.Cg-Tg(Tek-cre)12Flv/J), which is expressed in ECs during embryogenesis and adulthood [96]. EphA4^flox/flox^/Tie2::Cre male mice were bred to EphA4^flox/flox^ female mice to produce EphA4^flox/flox^ (WT) and EphA4^flox/flox^ /Tie2::Cre (KO) littermate pups. We confirmed that Cre activity was isolated to the vascular network by breeding the Tie2::Cre mice with double reporter labeled Rosa^mTmG^ mice (Jackson Lab) [46]. DNA isolation from tail samples were performed using 25mM NaOH incubation at 98°C for 1 hour, 15°C for 20 minutes then neutralized using 40 mM Tris HCL (pH 5.5). Genotyping of DNA was performed using polymerase chain reaction analysis and the following primers (1 μm): 5’-TGC TAA CAG GCA CTT AGA TCC C-3’ and 5’-TAA TTG TAA TCA GTG GGC GGG C-3’ to amplify floxed alleles; 5’- GCG GTC TGG CAG TAA AAA CTA TC-3’ and 5’- GTG AAA CAG CAT TGC TGT CAC TT-3’ to amplify Cre. The respective product sizes were 290 bp and 100 bp; DNA was amplified for 35 cycles (94°C for 1 minute, 51.7°C for 45s, 72°C for 1 minute) in a thermal cycler. Experiments comply with the ARRIVE guidelines for animal experimentation. Procedures related to animal use and care for this specific study was approved by the Virginia Tech Animal Use and Care Committee IACUC (protocols 15–063 and 14–044).

### Hindlimb Ischemia

Unilateral femoral artery and vein excision was performed by an investigator blinded to the experimental groups of 2–4 month old female WT or KO mice (5–8 mice/group), as described above. Inhalation anesthesia was administered using 1.5–2% isoflurane. Several drops of Bupivacaine (0.5%) were given near the incision prior to surgery. The state of anesthesia (lack of response to firm toe pinch, no body or whisker movement), rectal temperature (read-out from rectal monitor), breathing and coloration was monitored throughout the entire procedure. Animals were kept on a heating pad (homeothermic blanket system; Harvard Apparatus) and rectal temperature was monitored and recorded throughout the surgery. Body temperature was maintained at 37*C. Buprenorphine (0.1 mg/kg) was administer immediately following and for 2-days post-op. After dissection of the artery and vein from the nerve, ischemia was induced by twice electrocoagulation of the left femoral artery and vein, proximal to the superficial epigastric artery and vein and distal to sapheno-popliteal bifurcation. The artery was separated 1–2 mm between the cauterized ends at the end of the surgical procedure. Tissue outcome or appearance scoring was quantified and vascular perfusion was measured using non-invasive laser Doppler perfusion imaging prior to and immediately following surgery as well as at 3 and 7 days post-ischemia. No mice died during or following surgery nor were there any mice excluded from their respective groups. Animals were injected with Under 1.5–2% isoflurane and 37.0 ± 0.5°C perfusion imaging was performed at each time point, then ROIs were drawn to anatomic landmarks around the ischemic and control limbs. The valid pixel (VP) and mean pixel (MP) of ROIs from both left (L) ischemic limb and right (R) control limb left were obtained using moorLDI Laser Doppler Imager software, the ratios of ischemic (LVP*LM) versus control (RVP*RM) limbs were used to plot the time-course. Appearance score was determined by counting the number of necrotic toes at 7 days-post ischemia score. At the completion of the study, mice were euthanized by cervical dislocation under ketamine cocktail (Ketamine 100mg/kg, Xylazine 10mg/kg).

### Vessel Painting and pial collateral analysis

Vessel painting on post-natal murine pups was modified from previous studies [97]. Briefly, mice were injected s.c. with heparin (2,000 units/kg), and sodium nitroprusside (SNP, 0.75 mg/kg) five minutes prior to euthanization using an overdose of carbon dioxide. When breathing stopped, the chest cavity was opened and then cardiac perfused using a Gilson MiniPuls3 peristaltic perfusion pump (Gilson Scientific, Bedfordshire, UK). Using a continuous infusion, 6–10 ml of 1x phosphate buffered saline (PBS) containing 20 units/ml heparin was perfused to flush blood from the cerebrovascular system, then 10 ml DiI (0.01 μg/ml, Invitrogen)– 4% sucrose–PBS-heparin mixture was perfused to label the vasculature (0.7 ml/min for P1, 1.0 ml/min P7 and 2ml/min for P21 and adult flow rate), and finally, 4% cold paraformaldehyde (PFA) was perfused to fix the tissue. All reagents were filter sterilized and debris free. After perfusion, brains were carefully removed from the skull and placed in PFA overnight. Fixed brains were imaged at multiple image planes at 4x magnification on an upright fluorescence microscope (BX-51, Olympus America), using mosaic tile imaging from StereoInvestigator software (MBF, Williston, VT). Scaled mosaic images were imported into ImageJ, then the total number of intra- and inter-tree collaterals were identified between and within the MCA, ACA, and PCA artery branches and quantified using the counting tool in ImageJ on each mosaic image. Pial collateral diameters were also individually assessed on the scaled mosaic images using ImageJ by averaging three independent diameters along the collateral length.

### Measurement of hindlimb arteriole diameter and capillary density

Adductor muscle was harvested 7 days after induction of ischemia and fixed in 4% PFA overnight, then embedded in OCT and serial cryosectioned at 30 μM. Four sections at 300 μM apart were collecter per animal. Sections were then prepared and incubated with CD31 for the identification of endothelial cells and smooth muscle cells, respectively, to identify collateral arteries. Collateral artery diameter was measured using precalibrated microscope scale bars. Four sections were analyzed per animal by an investigator blinded to the groups and collateral diameters were quantified in 5 randomly selected low power (10×) fields per section; the mean value of these measurements was taken as a single data point for each animal. Capillary density was expressed as the ratio of CD31^+^ cells to myofibers. This measurement was determined in 5 randomly selected low power (200×) fields from each animal, and the average value taken and used as a single data point for each mouse.

### Histology

For immunostaining, whole mount, perfused-fixed tissue sections were blocked in 2% cold water fish gelatin with 0.1% Triton for 3 hours and incubated in primary antibody overnight in block at 4°C (rabbit anti-EphA4: 1/200 Santa Cruz). Sections were washed 4 times with 1X PBS and incubated with anti-rabbit Alexa Fluor 488-conjugated secondary antibodies (Molecular Probes, Carlsbad, CA) for 1h at RT. Whole mounts were counterstained with DAPI (1 μg/ml, Molecular probes, Carlsbad, CA) and mounted in Pro-Long anti-fade mounting solution (Molecular probes, Carlsbad, CA).

### Isolation and culture of endothelial cells

Post-natal day 1-5-old EphA4^flox/flox^ (WT) or EphA4^flox/flox^/Tie2::Cre (KO) pups were sacrificed by decapitation under anesthesia and whole brains were extracted and dissected using neural dissociation kit (Miltenyi Biotec, Auburn, CA). Four to five pups were used per group for each isolation. Single-cell suspension from fresh dissociated brain tissue was subjected to CD31^+^ magnetic beads and column separation, as per manufacturer instructions (MACS; Miltenyi Biotec, Auburn, CA). Cells were seeded into one well of a 6-well plate (Corning) pre-coated with Fibronectin (10 μg/ml; Corning, Corning, NY) in complete endothelial cell media with growth factors (Cell Biologics, Chicago, IL; M1166). Media was changed every 2 days until confluent then passaged using 0.25% trypsin/EDTA (Hyclone, Logan UT) and plated one T75 flask pre-coated with fibronectin. Cells were expanded and frozen or used for experimentation. Complete media changes were performed every 3 days until cells were confluent and passaged. Experiments were conducted on cells <10 passages.

### Proliferation, tube formation and migration assessment

ECs were plated in a 96-well plate containing EC complete media, pre-coated with 0.2% gelatin, at 20,000 cells/well. After 24 incubation, plates were removed and immediately assessed for proliferation by adding 10 μM bromodeoxyuridine (BrdU; Sigma Aldrich, St. Louis, MO). Following 1 h incubation with BrdU, cells were fixed with 10% buffered formalin and incubated in 2N HCl as previously described [76]. Cells were counterstained with DAPI (1 μg/ml) and analyzed under TRITC/DAPI filters on an inverted IX-71 Olympus epi-fluorescence microscope equipped with a digital XM-10 camera and Cell Sense software package (Olympus, Valley, PA). Four images per well were acquired and quantified as percentage of BrdU as previously described [29]. To assess tube formation, 15,000 cells were plated on a layer of approximately 60 μl solidified growth factor-reduced matrigel (Corning, Corning, NY) in EC base media without growth factors. Images were taken at 4x magnification using an IX-71 Olympus microscope. For migration assessment, we performed the scratch assay using 100,000 cells/well in a 24-well plate pre-coated with 0.2%gelatin, incubated overnight at 37°C; 5% CO_2_ in complete media. After 24 hours, a 200 μl pipet tip was used to scrape the cell monolayer in a straight line creating a scratch. Wells were washed twice with warm 1x PBS, then incubated in EC base media and incubated 4–48 hours. Wound healing or migration into the scratch was assessed at 4, 24 and 48 hours post-scratch. Images were taken at 4x magnification using an IX-71 Olympus microscope. All quantifications were performed using the measurement tools from Cell Sense software (Olympus, Valley, PA). In experiments assessing the role of PI3K inhibition on proliferation and migration, 50 μM LY294002 (Sino Biological Inc, PA) was added to EC culture media and DMSO added as a vehicle control at the time of plating or following wound scratch.

### qRT-PCR

Total RNA of endothelial cell cultures or fresh collateral zone tissue was isolated according to manufactures instructions using the RNeasy Mini Kit for total RNA extraction (Qiagen, Valencia, CA). RNA quantification was carried out by measuring absorbance with spectrophotometer ND-1000 (NanoDrop). RNA was reverse transcribed into cDNA with Im-Prom II Reverse Transcription System (Promega, Madison, WI). RNA samples were treated with DNase I (ThermoFisher, Waltham, MA) before reverse transcription. Each DNase reaction (1 μg RNA, 1 μL 10X DNase I buffer, 1 μL DNase I, 0.5 μL RNase inhibitor, up to 10 μL with water) was incubated at 37°C for 60 minutes before inactivation by addition of 1 uL 50 mM EDTA and incubation at 65°C for 10 min. The DNase-treated RNA samples were incubated with oligo (dT) 15 primer at 70 for 5 min. (250 ng RNA, 1 μL oligo (dT) 15, up to 5 μL with water) and chilled on ice for 5 minutes to allow annealing before reverse transcription. To prepare the cDNA samples, 15 μL reverse transcription mix (3.7 μL water, 4 μL 5X ImProm-II buffer, 4.8 μL 25 mM MgCl_2_, 1 μL 10 mM dNTPs, 0.5 μL RNase inhibitor, 1 μL ImProm-II reverse transcriptase) were added per 5 μL RNA sample and reverse transcription was performed using the following PCR scheme: 25°C for 5 min.; 42°C for 1 hour; 70°C for 15 min.

Differential gene expression was then assessed using qRTPCR. Primer sequences used were: GapdhF, 5’AAT GTG TCC GTC GTG GAT CTG A 3’; GapdhR, 5’AGA TGC CTG CTT CAC CAC CTT CTT 3’; Adam10F, 5’-ACGCTGGTGTTTTTGGTGTA-3’; Adam10R, 5’-AATTCTGCTCCTCTCCTGGG-3’; Adam17F, 5’-TCTTTGCTCTCAGACTACGACATCC-3’; Adam17R, 5’-CCACCACGACTCTCAAGTTTTGTG-3’; Ang1F, 5’-CGAATAACCAGTCAGAGGCAGT-3’; Ang1R, 5’-AGTAGGCTCGGTTCCCTTCC-3’; Ang2F, 5’-AGCAGATTTTGCATCAGACC-3’; Ang2R, 5’-GCTCCTTTCATGGACTGTAGC-3’; VegfF, 5’-GAAGTCCCATGAAGTGATCCAG-3’; VegfR, 5’-TCACCGCCTTGGCTTGTCA-3’; Vegfr1F, 5’-TTCGGAAGACAGAAGTTCTCGTT-3’; Vegfr1R, 5’-GACCTCGTAGTCACTGAGGTTTTG-3’; Vegfr2F, 5’-GGGACCTGGACTGGCTTTG-3’; Vegfr2R, 5’-CCGCATTCAGTCACCAATACC-3’; Mcp1F, 5’-TCACCTGCTGCTACTCATTCACCA-3’; Mcp1R, 5’-TACAGCTTCTTTGGGACACCTGCT-3’. For qRTPCR analysis, 7 ng cDNA per reaction were amplified using SYBR Green PCR Master Mix (Applied Biosystems, Foster City, CA). Expression changes were calculated using ΔΔCq values with reference to Gapdh internal control gene then calculated as relative expression compared to wild type samples.

### RNAseq Analysis

RNA-seq libraries were constructed according to Illumima protocol and sequenced with the Illumina Hiseq 2000. Using TopHat (version 2.0.3), all the 101bp pair-end reads were mapped to the mouse reference genome (GRCm38/mm10) [98]. Genome annotation files with GTF format for Known Genes were downloaded from UCSC. Reads per kilobase of transcript per million reads (RPKM) values were calculated for each gene using Cufflinks software (version 2.0.2) with default parameters [99] and normalized using quantile method. The files generated by the Cuffdiff program were then passed to the Cummberbund, an R package used to determine the significantly differentially expressed genes (P< 0.01) and to visualize the output. GO enrichment analyses were performed using DAVID functional annotation tools [100].

### Western Blot analysis

Protein of EC cultures was extracted by lysing cells in RIPA buffer (1% NP-40, 1% sodium-deoxycholate, 0.1% SDS, 0.15 M NaCl, 2 mM EDTA, and 0.01M sodium phosphate) in the presence of complete protease inhibitor cocktail (Roche) and phosphatase inhibitor cocktail 2 (Sigma). Supernatant was collected by centrifuging at 13000 g for 30 min at 4°C and the Lowry assay was used for determination of protein concentration (Pierce, Rockford, IL). Cell lysates (50 μg) were resolved on 8% SDS-PAGE gels and blotted onto PVDF membranes, blocked with 5% bovine serum albumin (BSA) in TBST buffer (20 mM Tris, 137 mM NaCl and 0.1% tween) then incubated in block overnight at 4°C with primary antibody against Ang1 (Rabbit, 1:1000 Rockland, Limerick, PA), MCP-1 (rabbit 1:1000 Sant Cruz Biotech, Santa Cruz, CA), or β-actin (mouse, 1:5000 Cell Signaling, Danvers, MA). HRP-conjugated secondary antibodies (Jackson laboratory) were applied to the membrane and developed as previously described [24]. Blots were quantified by densitometry using acquisition into Adobe Photo Shop (Apple, Cupertino, CA, USA) and analyzing by the NIH Image software (National Institutes of Health, Bethesda, MD, USA). The level of protein expression was normalized according to β- actin control levels. Samples were run in quadruplicate.

### Statistical analysis

Data was graphed using GraphPad Prism, version 4 (GraphPad Software, Inc., San Diego, CA). Student’s two-tailed t test was used for comparison of two experimental groups. For three or more groups, multiple comparisons were done using one-way and two-way ANOVA where appropriate followed by Tukey or Bonferroni test for multiple pairwise examinations. Changes were identified as significant if P was less than 0.05. Mean values were reported together with the standard error of mean (SEM).
